# A compound attributes-based predictive model for drug induced liver injury in humans

**DOI:** 10.1371/journal.pone.0231252

**Published:** 2020-04-15

**Authors:** Yang Liu, Hua Gao, Yudong D. He

**Affiliations:** 1 Genome Analysis Unit, Amgen Research, Amgen Inc., South San Francisco, California, United States of America; 2 Molecular Engineering, Amgen Research, Amgen Inc., Cambridge, Massachusetts, United States of America; University of Colorado Denver Skaggs School of Pharmacy and Pharmaceutical Sciences, UNITED STATES

## Abstract

Drug induced liver injury (DILI) is one of the key safety concerns in drug development. To assess the likelihood of drug candidates with potential adverse reactions of liver, we propose a compound attributes-based approach to predicting hepatobiliary disorders that are routinely reported to US Food and Drug Administration (FDA) Adverse Event Reporting System (FAERS). Specifically, we developed a support vector machine (SVM) model with recursive feature extraction, based on physicochemical and structural properties of compounds as model input. Cross validation demonstrates that the predictive model has a robust performance with averaged 70% of both sensitivity and specificity over 500 trials. An independent validation was performed on public benchmark drugs and the results suggest potential utility of our model for identifying safety alerts. This *in silico* approach, upon further validation, would ultimately be implemented, together with other *in vitro* safety assays, for screening compounds early in drug development.

## Introduction

Despite the current advancements of many scientific and technological factors such as genetics, chemistry and protein engineering, the efficiency of drug research and development (R&D) has not risen intended [1]. High-profile drug attrition substantially hinders R&D productivity, with unacceptable safety often a reason for failures [2]. Therefore, identifying potential risks due to adverse drug reactions (ADRs) as early as possible in drug development pipeline and prospectively predicting their likelihood as accurate as possible in humans is one of the most important and challenging tasks for the pharmaceutical industry.

Prior to drug trials in humans, extensive preclinical studies are conducted to examine efficacy and toxicity [3]. In addition to animal models, alternative translational approaches, especially *in silico* computational models, are widely used to refine drug development design, and reduce the size and duration of clinical series [4]. Various *in silico* efforts have been reported with predictive modeling based on drug properties [5], drug-ADR connections [6], drug-drug similarities [7], drug-drug interactions [8], or drug-target effects [9, 10], in order to detect pharmaceutical-related toxicities for different phenotypes [11, 12], based on different source of safety data [13, 14], suggesting its fundamental role in toxicity prediction.

Of various real-world adverse drug event knowledge bases, FAERS is very comprehensive. It is a spontaneous reporting system that collects and houses adverse events reported by clinicians, patients and drug manufacturers from all over the world. It details other capturable information such as drug exposures, disease indications, demographic information and clinical outcomes. FDA started collecting these data in 1968 and currently there are over 5 million accumulated reports, with a rapid growth rate every year [15]. FAERS brings a rich opportunity to detect novel post-marketing ADRs, reveal populations that are not well represented in clinical trials, and better serve early development pipeline by studying lessons and extracting knowledges from post-marketed drugs. Numerous studies have demonstrated the significant power of FAERS to discover pharmacovigilance signal and its influence to premarketing research phases [16–18].

Among all the causes of regulatory actions, including denial of approval, black box warnings and withdrawal from the marketplace, DILI is the most frequent cited reason [19]. Wysowski [20] *et al* found that 12 out of 76 drugs (16%) approved between 1969 and 2002 that required a black box warning or removed from the market are attributable to DILI. Similar studies was performed by Lasser *et al* [21]. Whereas there are liver signals that escape detection during current standard *in-vitro* and *in vivo* tests, the risk of false positives may lead to unnecessary attrition as well. While DILI is a multifactorial phenomenon with all mechanisms still not fully understood, an *in silico* test that bases on compound physicochemical and structural attributes is believed to be able to capture most of the unknown mechanism-independent factors, and to provide insights into the complex dynamics of liver injury as early as in the screening stage.

Numerous computational models have been developed in the past to predict toxicological properties of a given compound that cause DILI. However, such efforts have limited utility so far due mainly to the fact many of these models cannot be applied to novel compounds. A number of papers have reported notable performance for predicting ADRs based on similarity calculation by considering compound structural similarities [22–24], target structural similarities [25], side effect similarities [26] or a combination of all [7, 27]. However, intrinsic limitations of similarity-based methods are that certain evaluations of similarities cannot be fully captured for novel compounds. For example, the International Classification of Diseases (ICD) code has not been annotated or the target has not been determined. Furthermore, the similarity calculation can explain how similar of two compounds in terms of a particular property, but it is not interpretable in terms of what is the underlying influence of this property to each compound. Other papers assemble comprehensive features that reflect variations among compounds, and then apply these features into different modeling systems to predict toxicity [5, 28, 29], but most of these approaches focus on approved and withdrawn drugs, either with limited tests on or nonapplicable to compounds that still in early development phase.

In consideration of aforementioned points, we proposed a novel predictive model specifically designed to identify drugs with high likelihoods related to DILI, based on selected features of their physicochemical and structural properties. This way, it is possible to readily apply the model to compounds as early as possible during the development. Based on cross-validation, the model demonstrated a robust performance with a reasonable level of sensitivity and specificity. To test the potential application of this model in early-stage compound screening, all the internal attrition small molecules due to DILI are assessed, and the model shows good prediction ability and feasible utility. In addition, we validated our model on an independent set of benchmark drugs, suggesting its potential for real world detection of ADR signals.

## Results

**Fig 1:**
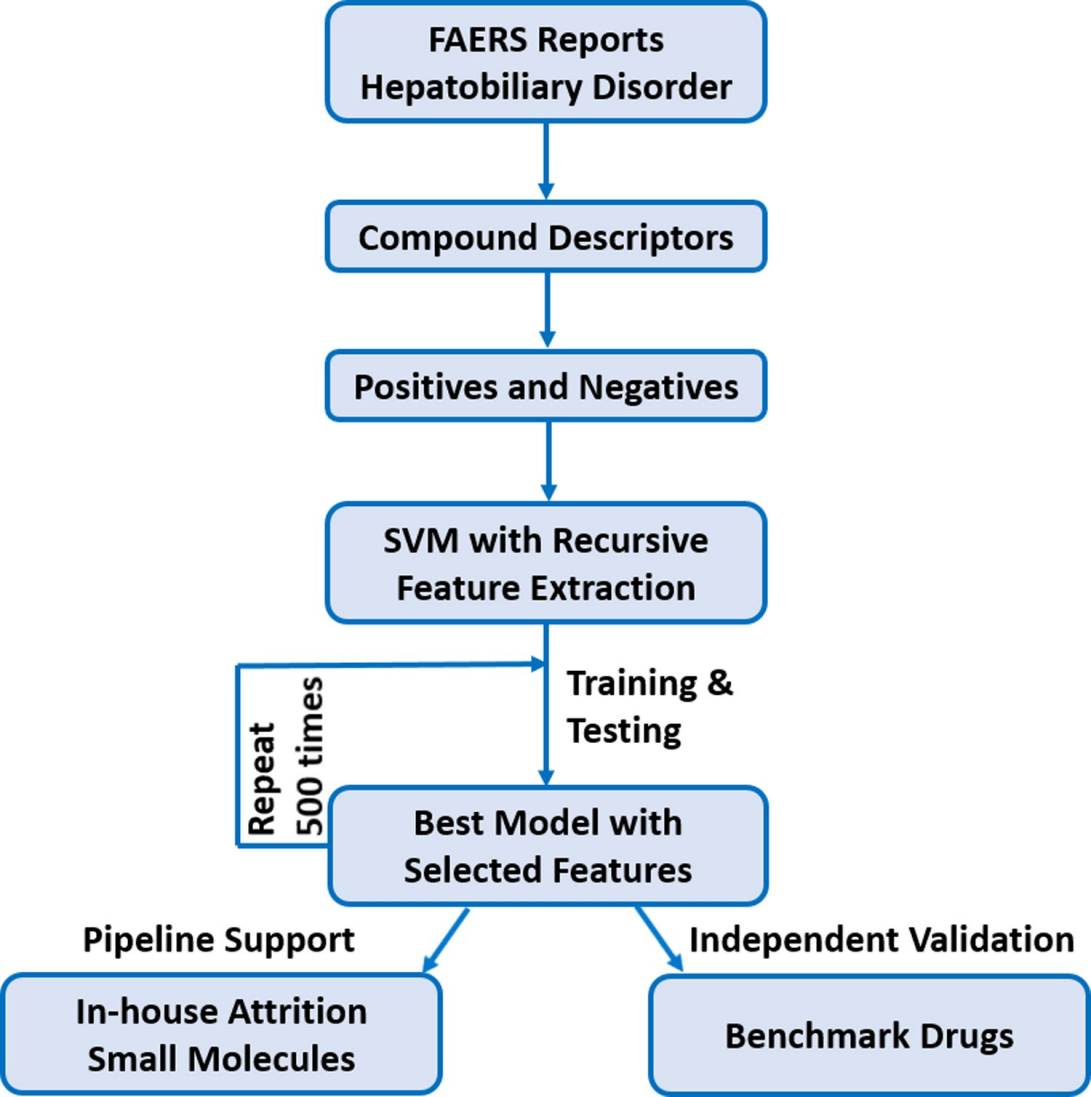
Flowchart of the SVM model.

Details of our analytical approach and data source are described in Methods. Briefly, we work only on FAERS reports of hepatobiliary disorder. All drugs that are either significantly involved in these reports (positive) or totally clean (negative) are included as a set of training compounds. A comprehensive set of 818 descriptors (including physicochemical and structural properties) were generated for each of positives and negatives using our in-house cheminformatics tool. The training compounds were then fit into a Support Vector Machine (SVM) with recursive feature extraction. The SVM model with optimized parameters and selected features was then applied to any future use scenarios. The flowchart of this method is illustrated in Fig 1.

### Signal detection from FAERS

The overall data collection (2004–2015) resulted in 2,542,867 individual reports, involving a total of 1,208 unique drugs in 13,665 unique events after mapping drug names to DrugBank (https://www.drugbank.ca/). Since our focus was on DILI in this study, we were particularly interested in those reports that are liver related. All the events that are reported in Preferred Term (PT) in MedDRA system (https://www.meddra.org/) were aligned to their hierarchical System Organ Category (SOC) terms and the total number of reports from each individual PT terms were accumulated correspondingly.

**Fig 2:**
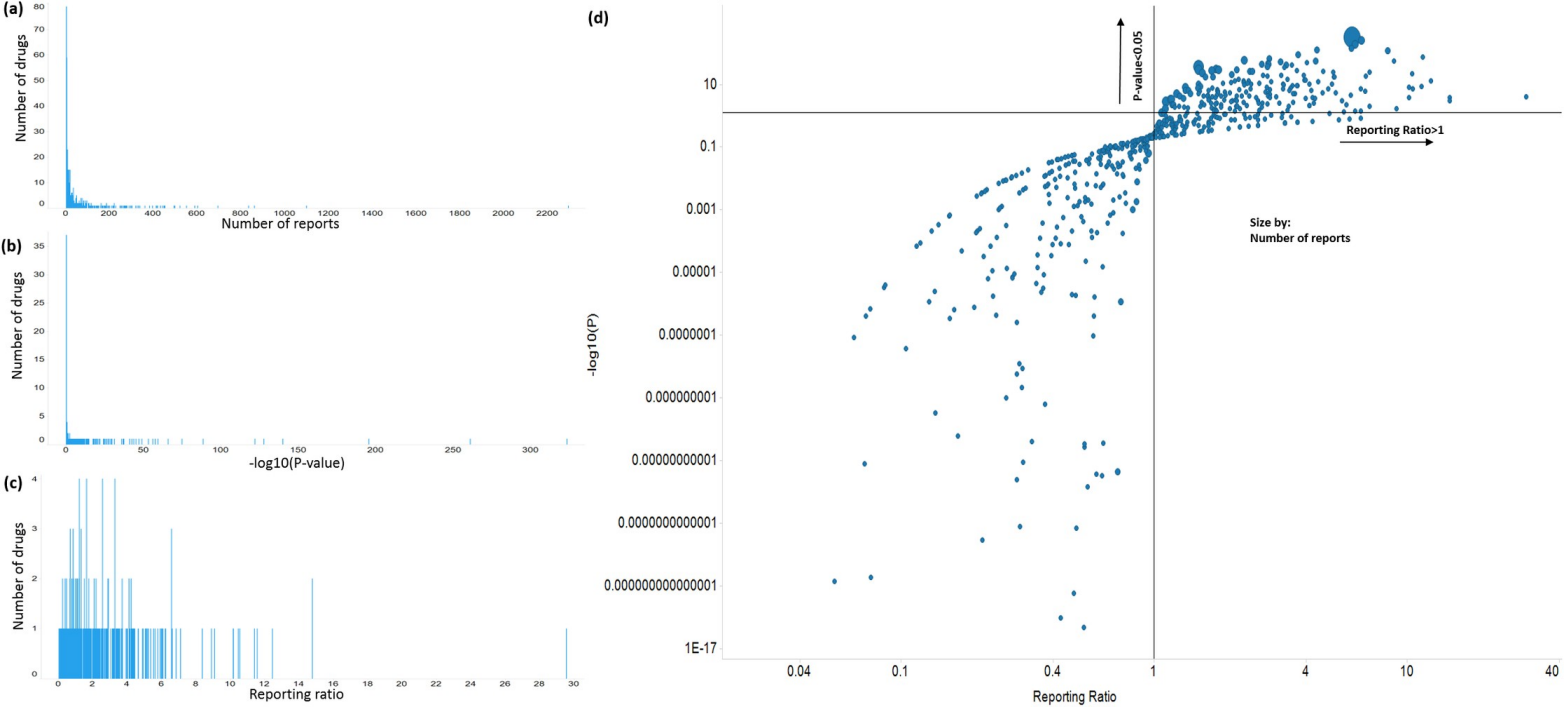
Statistical association between drugs and “hepatobiliary disorder” reports. (a) Distribution of number of reports. (b) Distribution of–log10(P-value). (c) Distribution of reporting ratio. (d) Relations of reports number, -log10(P-value) and reporting ratio.

Following the procedure described in the Methods, we identified a total of 620 drugs that have at least one report as primary suspect involving end-organ of “hepatobiliary disorder”. The total number of reports for each drug were applied to calculate whether this drug has strong association with hepatobiliary disorder, based on both Relative Reporting Ratio (RRR) and corresponding P-value, see Methods. The distribution of reports number, RRR and -log(P-value) are shown in Fig 2.

We finally narrowed down 155 drugs that were regarded as “positives” for liver-related events, with each had at least 13 reports in hepatobiliary disorder, RRR > 1, and P-value < 0.05. Meanwhile, we selected 164 drugs that were regarded as “negatives” for liver-related events, with each had no single report in hepatobiliary disorder and have been in market for at least 10 years.

### Compound-based attributes

For each of these 155 positive compounds and 164 negative compounds, we generated a comprehensive molecular profile based on their physicochemical and structural properties. The tool used for generating the molecular profile includes the most common descriptors in ADMET modeling are developed in Amgen and implemented in MOE (https://www.chemcomp.com/Products.htm). In total, 818 descriptors were used for such a profile. Ranking order them according to their relevance to the DILI predictor in a univariate manner is possible. Rather, we treated them equally and used all 818 descriptors as input for feature selection and predictive model building.

### Model performance characterization

**Fig 3:**
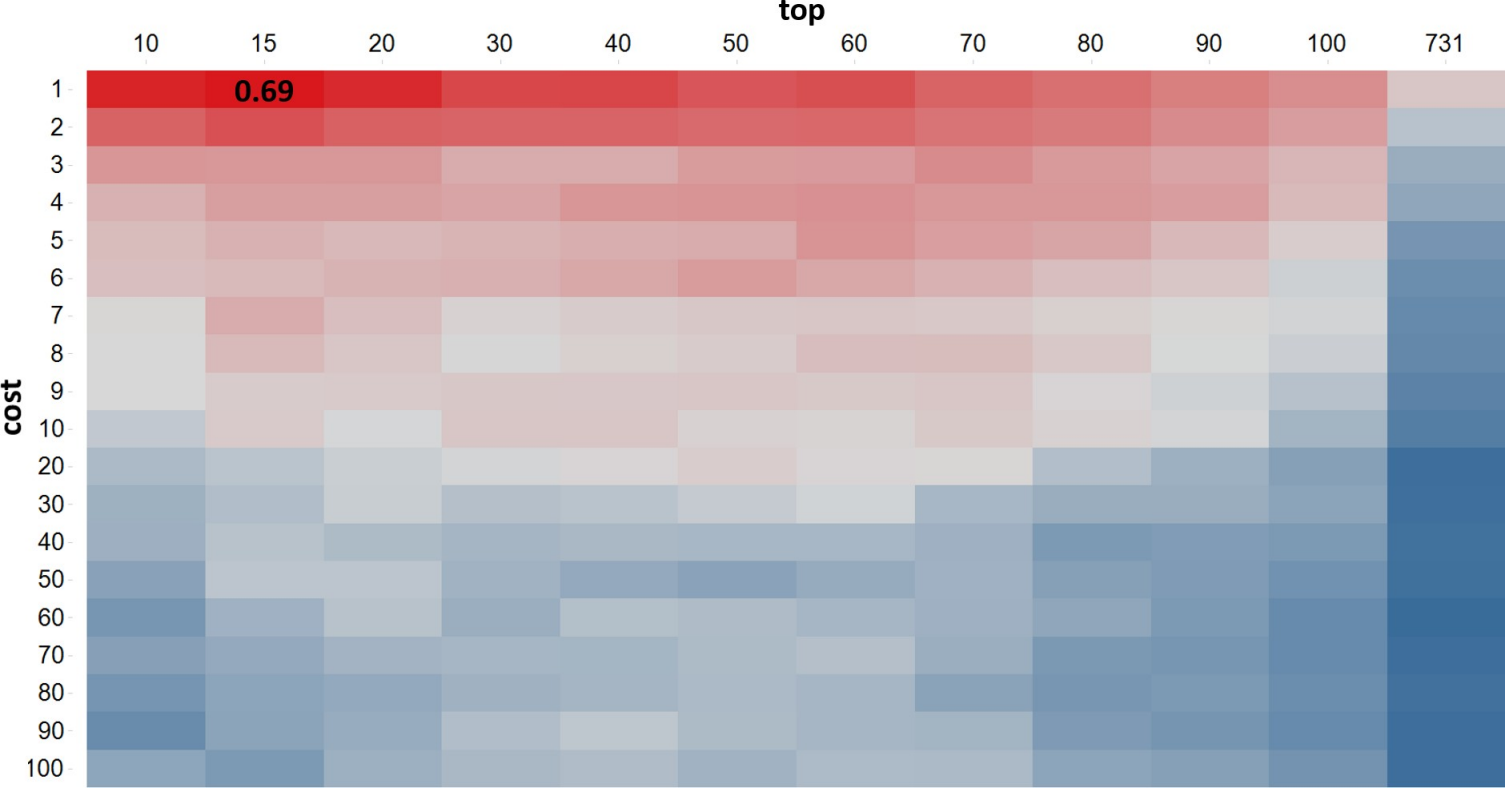
Heatmap of AUC under different *cost* and *top* settings. Each row is individual *cost* values while each column represents individual *top* values, and each cell is the averaged AUC over 500 training sets. The best averaged AUC is 0.69, when *cost* = 1, *top* = 15.

We built SVM models with recursive feature extraction using the input of 818 descriptors from each training set containing equal number of positive drugs and negative drugs. In total, we examined 500 such training sets, each of them with the same positive set (155) and randomly selected negatives with the same number of positives (155) from the entire negative compound pool (164). For every compound selected in any training set, we calculated 818 descriptors using our internal tool, see Methods. Each of these 500 training sets resulted in multiple SVM models, with each individual model was trained and tested under different parameter combinations of *cost* and *top*. Each parameter combination was evaluated by averaging their performance across these 500 training sets, and the best combination of *cost* and *top* were selected when the averaged area under curve (AUC) got maximized over 500 times, which is 0.69 when *cost = 1*, *top = 15*, see Fig 3. In the circumstance of *cost = 1*, *top = 15*, the averaged performance over 500 training sets are: Sensitivity = 70%, Specificity = 70%, False Positive Rate (FPR) = 30%, False Negative Rate (FNR) = 30%, Accuracy = 70%, Positive Predictive Value (PPV) = 71%. Correspondingly, the averaged probability to distinguish positive and negative is 0.48, which illustrates the possibility of a compound to be positive, with higher means larger possibility and lower otherwise.

### Feature frequency distribution

**Fig 4:**
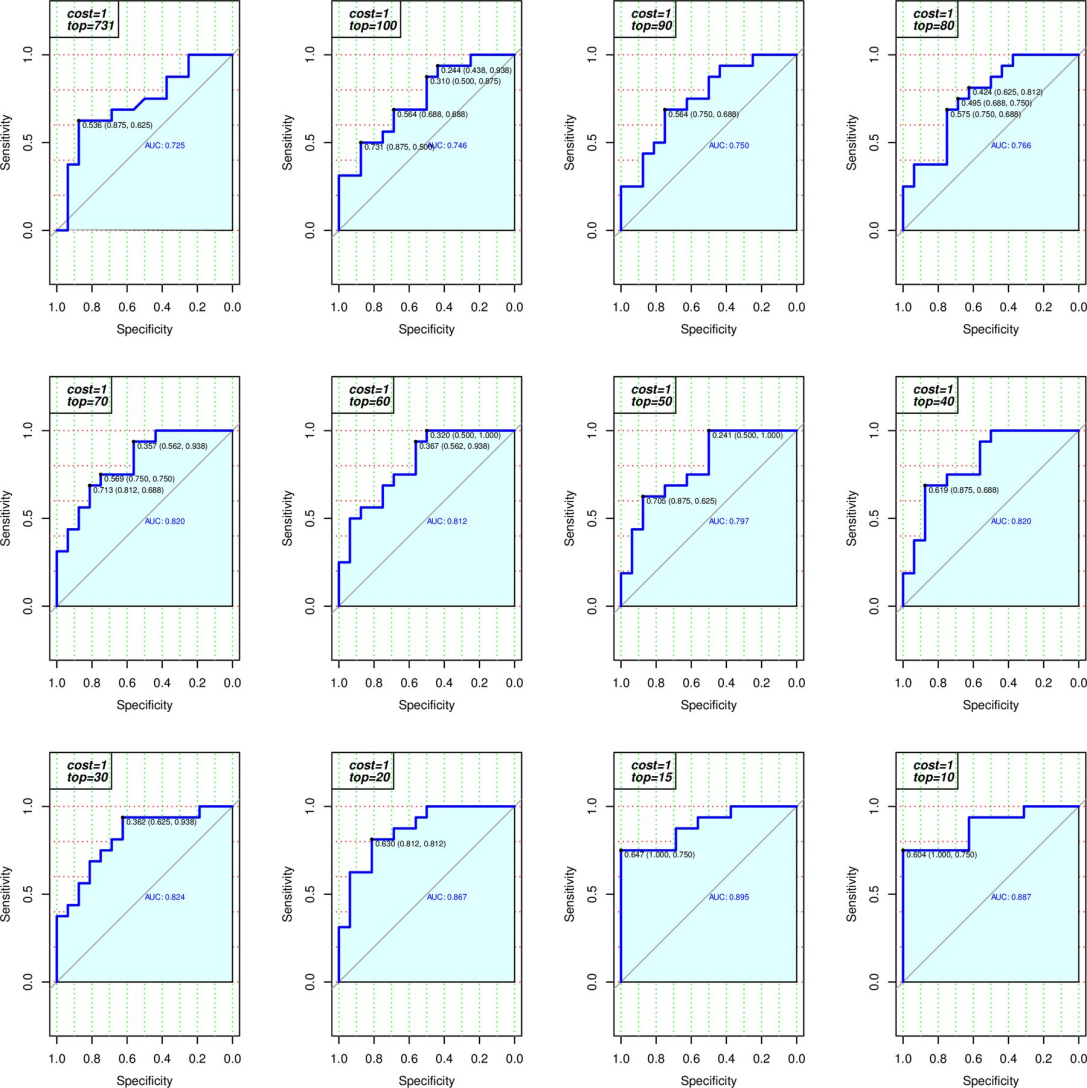
ROC from one of the training set. These ROC curves are obtained when fixing *cost = 1* and iteratively decreasing *top* values from 731 to 10. In each ROC plot, the AUC value is calculated, and the corresponding sensitivity and specificity are marked.

It is expected that some of these 818 molecular descriptors are highly redundant. By recursively eliminating correlated features, the performance of the model improved, as seen in Fig 4, which shows a series of the receiving operating characteristic (ROC) curves from one of the training sets by fixing *cost = 1* but iteratively decreasing *top* values from 731 to 10.

**Fig 5:**
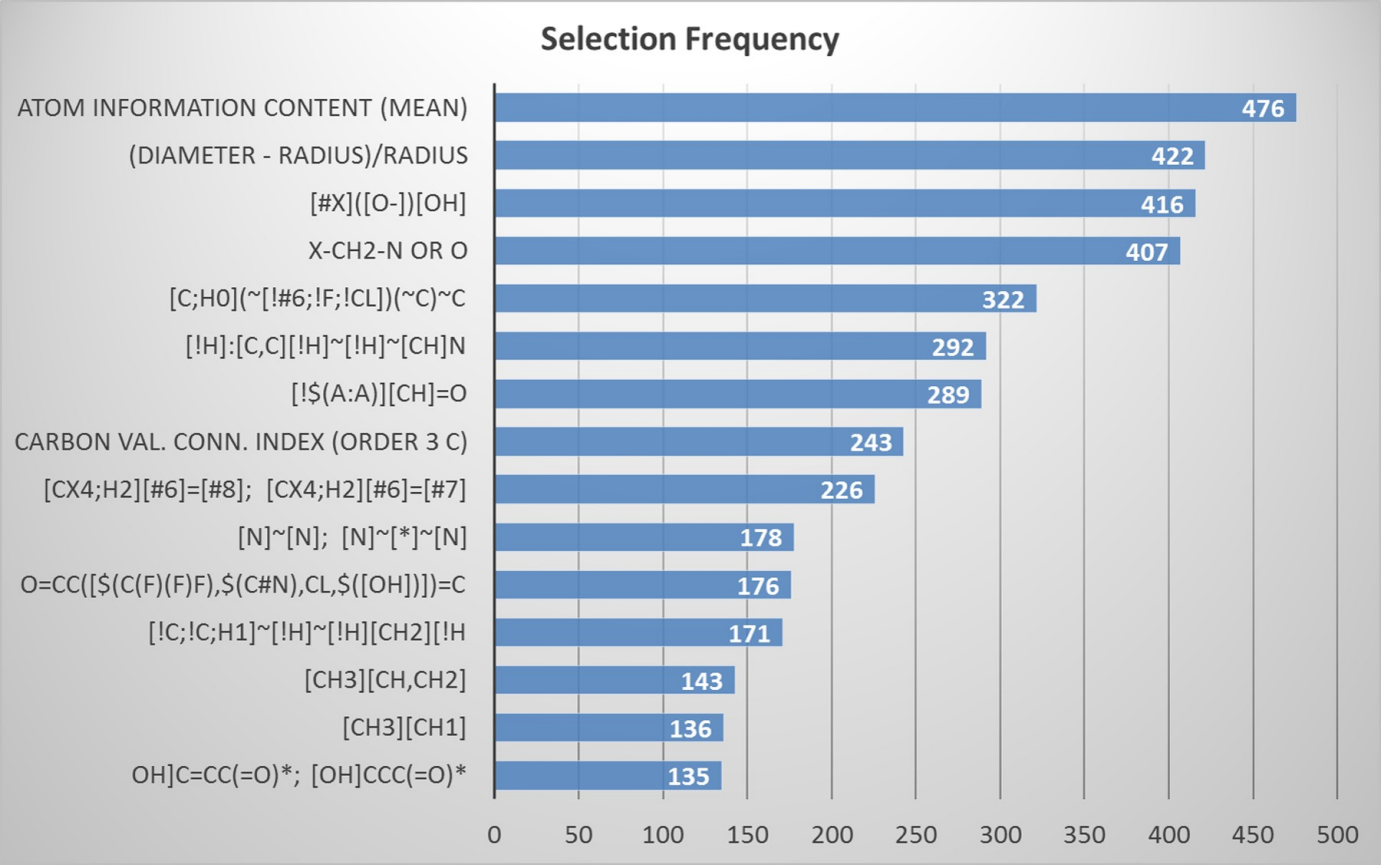
Top 15 features selected by SVM model. The features are ranked by selection frequency descending order, while frequencies are marked at the right side of the bar.

It is noted that on average, the best performance can be achieved when the number of top features is within the vicinity of 15 (*top = 15*). However, the top 15 features from each of the 500 training sets are not always identical. Amongst the union set of all feature candidates from 500 trials, we ranked them by their frequency of presence. We then selected the top 15 features with the highest frequency for the final model to be used for independent validation, see Fig 5. Note that the top ranked feature (atom information content) appeared 476 times (95.2%) and the 15^th^ ranked feature appeared 135 times (27%) out of 500 trails respectively.

### In-house small molecules testing

As a realistic check of the final predictive model trained based on FAERS, we systematically queried 4,400 internal small molecules in Amgen compound database, and fitted them into our trained final SVM model, and outputted the likelihood of each compound being positive. Since most of these internal compounds do not involve in any development pipeline and lack of confirmed and solid evidence of its liabilities to cause DILI, it would be challenging to directly evaluate all these internal compounds against our predictive model. In order to get an estimation, we have performed the following two steps. As the first step, we compared the predictive model with test results from one of Amgen’s liver safety panel—human Bile Salt Export Pump (BSEP) assay, so that we could obtain an approximate performance estimation of our predictive model. Specially, we first compared the IC50 value of these compounds tested through BSEP assay with the likelihood values predicted by the final SVM model, to see how concordant they are. As the second step, we then tested our model against all internal small molecules that have been terminated either in preclinical and clinical stages due to liver related issues, to get a more accurate and refined estimation of the model performance.

Of those 4,400 compounds, 3,023 of them can be conclusive by BSEP, either positive (IC50< = 25) or negative (IC50> = 100), and 1,377 of them are not determinate (25<IC50<100). 2,399 out of those 3,023 compounds have the same output from both BSEP assay and SVM model, which results in a concordance rate as high as 79%, the remaining 624 compounds are in disagreement between these two systems, leading to a discordant rate of 21%. Even we have *in vitro* (BSEP) and *in silico* (SVM model) results available, the gold-standard criteria for DILI liabilities cannot be confirmed till preclinical or clinical tests are completed for each candidate.

To further evaluate the SVM model performance, we made manual inquiry into those 4,400 internal small molecules to identify the ones terminated in different phases of our development pipeline due specifically to liver related issues. As the result, we identified 12 such small molecules, as summarized in Table 1.

**Table 1:** In-house small molecules testing.

AMG ID	BSEP		SVM Model		Comments
	Output	IC50	Output	Probability	
A	Pos	9	pos	0.75	Killed in Phase I due to elevated transaminases
B	Inconclusive	44	pos	0.76	Killed in RPT, mild to minimal hepatic lipidosis
C	Inconclusive	89	pos	0.73	Killed in PT, inflammations found including liver, repurposed for oncology
D	Inconclusive	39	pos	0.71	Killed in RPT, hepatic vacuolation
E	Inconclusive	57	pos	0.61	Killed in RPT, hepatic vacuolation
F	Inconclusive	35	pos	0.60	Killed in RPT, hepatic lipidosis and glucokinase translocation
G	Inconclusive	29	pos	0.60	Inactive in RPT, hepatic/biliary injury
H	Inconclusive	68	pos	0.50	Inactive in RPT, hepatic/biliary injury
I	Pos	23	neg	0.34	Killed in Phase I, elevated transaminases
J	Pos	25	neg	0.28	Killed in Phase I, elevated transaminases
K	Pos	19	neg	0.29	Inactive in RPT, hepatocellular degeneration, necrosis and loss
L	Neg	100	neg	0.45	Killed in PT, hepatobiliary injury

BSEP, bile salt export pump; SVM, support vector machine; RPT, research product team; PT, project team.

For these 12 terminated compounds due to liver-related issues, our trained SVM model predicted 8 in positive category and 4 in negative class, leading to a sensitivity of 67% (8/12) and FNR of 33% (4/12). The most promising evidence is from Compound A, as its development was halted during Phase I human trials due to an observed transaminase elevation [30]. Noticeably, for Compound A, there was no preclinical evidence of liver injury in all animal studies including mice, rats, hamsters, rabbits and non-human primates. Additionally, all assay-based tests, including *in vitro* cytotoxicity, mitochondrial toxicity, covalent binding, and reactive metabolite formation data, did not suggest a plausible mechanism for liver injury in humans. However, transaminase elevations occurred in 5 of 8 healthy volunteers during Phase 1 trial of twice daily dosing with 100mg/dose, and returned to normal upon cessation of Compound A treatment. Compound A was consequently found to involve in multiple hepatocellular transporters, including BSEP. According to our predictive model, the probability of Compound A to cause liver injury is as high as 75%, demonstrating the powerful foresight of this *in silico* SVM model for DILI that is undetected during preclinical phases with animal studies.

The remaining 7 positives predicted by SVM were all with high probabilities and were all stopped for development during pre-clinical stages due to various liver-related safety issues. Interestingly, none of them were conclusive from BSEP assay results, which plays significant role in current liver toxicity safety panel assessment. This demonstrates the potential power of the *in silico* model in terms of identifying liver toxicity related safety concern in very early stage once the candidate compound is made and its molecular profile is available. For positive candidate compounds predicted by this *in silico* model, we can then subject them to selected low-throughput assays to further confirm the liver-related injuries and study the underlying mechanisms.

### Independent validation

To further valid the *in silico* model we developed, a separate set of 285 benchmark drugs were selected and annotated for their known association with DILI and tested independently with this final SVM model. Of these 285 drugs, 265 were curated internally by liver experts in reference with available literature. We found that 63 of them have confirmed connections with liver injury, either from public literature or previous studies, and the rest 222 of them are considered as negative. When applying our model to these 285 benchmark drugs, we achieved a sensitivity of 63% (40 out of 63), specificity of 66% (146 out of 222), FPR of 34% and FNR of 37%.

This predictive model is sensitive in detecting drugs that are withdrawn due to liver related issues. For example, Tolrestat is an aldose reductase inhibitor which was approved for the control of certain diabetic complications (https://www.drugbank.ca/drugs/DB02383). While it was approved in several countries, it failed a Phase III trial in the U.S. due to liver toxicity and never received FDA approval. It was discontinued by Wyeth in 1997 because of the risk of severe liver toxicity and death. Its probability for causing DILI is 0.74 predicted by our model. Another withdrawn drug we successfully predicted is Ticrynafen (https://www.drugbank.ca/drugs/DB04831), a diuretic drug with uric acid-lowering (uricosuric) action, formerly marketed for the treatment of hypertension. It was withdrawn in 1982, shortly after its introduction to the market, after case reports in the United States indicated a link between the use of ticrynafen and hepatitis [31]. Its probability to be positive is 0.69 according to our model. Other terminated drugs with known associations with liver injury and predicted as positives are Pirprofen (withdrew in 1990, probability = 0.66), Fenclozic acid (withdrew in 1970, probability = 0.60), and Benoxaprofen (withdrew in 1982, probability = 0.59). All these examples suggest the powerful predictive ability of this SVM model trained based on FAERS data to detect DILI.

Our predictive model is also helpful to recognize drugs that have contraindications of liver deficiency conditions. Take Chlordiazepoxide as an example, its use should be avoided in individuals with severe liver deficiencies such as hepatitis and liver cirrhosis. Zafirlukast is also contraindicated in patients with hepatic impairment including hepatic cirrhosis, it has a probability of 0.70 to cause liver problems. Other such positive drugs include but not limited to Docetaxel, Paclitaxel and Temsirolimus. All these positive discoveries give us more complete perspectives about the potential toxicological effects of a candidate compound, it also gives us better strategy to apply when potentially unsafe drugs cannot be avoided.

## Discussion

Accurate and timely prediction of drug induced liver injury remains a challenging research topic with a great potential impact in drug R&D. A number of efforts have been made to build *in silico* models that can predict DILI [32–34]. Since the datasets and feature selection vary, a direct comparison between these methods and our approach may be inaccurate. However, the common drawback of these methods is they have limited utility in drug development especially at the early stages, which is also the motivation to build this model, i.e., to provide a translational tool of toxicity review of DILI liabilities for new compounds that entering our pre-clinical pipeline as early as possible.

We demonstrated that evaluating profiles of compounds including physicochemical and structural properties can capture mechanism-independent attributes, prune the prediction as accurate as possible and identify potentially important liabilities as early as possible. By building this SVM model with recursive feature extraction based on hepatobiliary disorder reports in FAERS, we characterized its performance with reasonable sensitivity and specificity after cross validation. In our initial estimation, we found acceptable sensitivity of the model to uncover in-house attrition small molecules due to liver related issues in both preclinical and clinical studies. In a following independent test on benchmark drug molecules, we illustrated the power of the model to reveal withdrawn drugs with liver issues and drugs having contraindication with liver deficiencies.

The current version of model is based solely on compound attributes. In the future, we may be able to integrate compound’s target and downstream influences into one predictive model. By expanding our current underlying principle, which assumes that compounds with similar chemical structure exhibit similar profile for adverse hepatic reactions, we would further expect that compounds with similar *in vitro* protein binding profiles and downstream pathway profiles tend to exhibit similar profile for side effects as well.

In summary, we performed an extensive mining of available post-marketed DILI reports housed in FAERS. From these filtered data we identified positives and negatives, and we then developed training sets of drugs to build a SVM model using compound chemical, physical and structural properties as input. An internal attrition test and another independent validation show a great potential of this *in silico* model to identify possible safety concerns. This approach, if further validated in future studies, would be implemented in as early as in lead development stage to screen compounds for liver-related liabilities.

## Methods

### FAERS data source and pre-processing

The data used in this analysis covers adverse events from January 2004 to September 2015, which comprises millions of reports and offers the opportunity to examine the legacy data (2004Q1-2012Q3) as well as new events (2012Q4-present). Of the drugs and events included in each report, we only considered drugs reported as “primary suspect” and assumed that all events involved in this report come from the primary suspect drug.

In FAERS, all suspect phenotypes are coded using standard terminology of MedDRA, but all drug names are entered in freestyle including generic names and trade names with misspellings or names together with dose/purchase information. We further mapped the primary suspect drug name with DrugBank, and limited our analysis to drugs that can have an exact match with DrugBank.

In order to calculate any following statistical analysis, all drug-event pairs found between January 2004 and September 2015 were first constructed. We then consolidated each drug-event combination by adding their reported numbers together. A summarized matrix was finally built, which constitutes all unique drug-event pairs and their corresponding total number of reports during the time span.

### Statistical association

Using above summarized data, we constructed two-by-two contingency tables for each combination of drug-event pairs, see Table 2. To capture drug-event association strengths, we included two pre-calculated measures of disproportionality analysis to identify drug-event pairs that are strongly associated.

**Table 2:** Two-by-two contingency table for drug-event pairs.

	Reports for Event of Interest	Reports for All Other Events	Total
Reports for drug of interest	a	b	a+b
Reports for all other drugs	c	d	c+d
Total	a+c	b+d	a+b+c+d = N

For the Relative Reporting Ratio (RRR) [35] calculation, we used:

$$RRR=\frac{a*N}{\left(a+c\right)*\left(a+b\right)}$$

We then determined the statistical significance of the RRR by Fisher exact test.

### In-house small molecules

We assembled a set of 4,400 compounds available for internally prospective testing, in which one of Amgen’s liver safety panels—BSEP assay readouts are available. To have an initial systematical evaluation about activity prediction across these two platforms, determinations of positives/negatives based on IC50 values from BSEP are matched towards the compound liabilities assessed by the predictive model. Additionally, a narrowed down investigation about attrition small molecules due to liver related issues were further performed to verify the potential ability of this predictive model to support drug development pipeline. This results in 12 compounds, terminated in either pre-clinical or clinical stages.

### Benchmark compounds

The literature was reviewed for compounds known to interfere with liver injury [36]. A collection of benchmarks with known liver liability but not included in training set were then manually picked, which results in 63 positive drugs that are either marketed or withdrawn, to predict model’s sensitivity. Correspondingly, drugs with no known DILI associations, were selected from this benchmark compound pool [36] to test model’s specificity. By excluding compounds that already contributed in training, also excluding those that have any reports in hepatobiliary disorder in FAERS, we finally got 222 negative compounds.

### Compound descriptors

Compound descriptors are either numerical or categorical values computed from a compound’s structural SMILES, using software MOE. We selected a comprehensive 818 descriptors, which are the most common used descriptors in Amgen ADMET modeling. These include but not limit to physical properties (e.g., Molecular Weight, slogP), atom counts and bond counts (e.g., atom information content), pharmacophore features (e.g., a_acid), adjacency and distance matrix descriptors (e.g., diameter, petitjean) or sub-structure information.

### SVM with recursive feature extraction

The positive sets are drugs that have at least 13 reports in hepatobiliary disorder, with RRR>1, P-value<0.05, which results in 155 of them. A much larger set of negatives with total number of reports equal to 0 is expected since lots of drugs do not have any reports in hepatobiliary disorder at all. However, some of the drugs are too new to be evaluated. We therefore introduced “year of patent” as another parameter to narrow down negatives. Negatives need to have no reports in hepatobiliary disorder and its year of patent has to be at least 10 years since now, which means negatives are drugs that keep clean in hepatobiliary disorder for more than a decade, which results in 164 of them.

To keep the training set perfectly balanced, we repeatedly selected a random sample of 155 compounds from the 164 negatives and used it together with the positives to re-train the model. The random selections are done through an undersampling method. We repeat these trials 500 times to obtain 500 different training sets, with each individual set is used with SVM model separately. For each individual data set, we randomly select 90% of both positive and negative compounds for training and use the remaining 10% of the compounds to evaluate the model.

The R “e1071” package was used to perform SVM, while “OmicsMarkeR” package was used to recursively extract features. There are 2 parameters to tune: i) cost value *cost*, which is a trade-off between error penalty and margins, ii) number of features *top*, which indicates how many top ranked features are selected. For one particular training set, multiple SVM models were built for different combinations of these 2 parameters, with corresponding ROC curve, AUC value, sensitivity, specificity, FPR, FNR, accuracy, PPV and probability (threshold to separate positive and negative) calculated. Each of the 500 training set was repeated above steps to get all combinations of parameters tested.

For each unique combination of *cost* and *top*, a summarized performance over 500 training set was averaged. To get one final SVM model in any future testing, the combination of *cost* and *top* with the best averaged AUC value was selected as the final parameters, the corresponding averaged probability was considered as the probability to separate positives and negatives, and the averaged performance (sensitivity, specificity, FPR, FNR, accuracy, PPV) was calculated as the performance of our predictive model.

Even fixed *cost* and *top*, the top features selected by each training set is still not exact the same. We first got all features that equal to *top* in each of the training set, ranked them by the frequency of their selection by these 500 training sets, and only chose the *top* features that with the highest frequency. These refined *top* features were used in our final SVM model for any future analysis.

## Supporting information

S1 Data (XLSX)
